# The Progress of Next Generation Sequencing in the Assessment of Myeloid Malignancies

**DOI:** 10.4274/balkanmedj.galenos.2018.2018.1195

**Published:** 2019-02-28

**Authors:** Fulya Öz Puyan, Serhan Alkan

**Affiliations:** 1 Department of Pathology, Trakya University School of Medicine, Edirne, Turkey; 2 Department of Pathology and Laboratory Medicine, Cedars-Sinai Medical Center, California, USA

**Keywords:** Myeloid neoplasia, next-generation sequencing, somatic mutation

## Abstract

The introduction and advances on next-generation sequencing have led to novel ways to integrate simultaneous assessment of multiple target genes in routine laboratory analysis. Assessment of myeloid neoplasms with targeted next-generation sequencing panels shows evidence to improve diagnosis, assist therapeutic decisions, provide better information about prognosis, and better detection of minimal residual disease. Herein, we provide information for application and utilization of next-generation sequencing studies with a focus on the most important mutations in acute myeloid leukemia, myelodysplastic syndrome, myeloproliferative neoplasms, and other myelodysplastic / myeloproliferative neoplasms in order to integrate them into the daily clinical practice.

Next-generation sequencing (NGS) technology can be of great utility in the clinical assessment of various neoplasms. NGS can be rapidly integrated into the diagnostic laboratories and merged into current clinical practice. However, bioinformatics and genetic code technology and language are difficult to understand for practitioners who are not familiar with molecular biology. We will review the recent developments in NGS, technical progress, and focus on the approach of diseases affecting the myeloid lineage, including acute myeloid leukemia (AML), myelodysplastic syndrome (MDS), chronic myeloproliferative diseases (CMPDs) such as polycythemia vera (PV), essential thrombocythemia (ET), primary myelofibrosis (PMF), atypical CMPD, chronic myelomonocytic leukemia (CMML) and chronic eosinophilic leukemia (CEL) and mast cell disease.

Due to the increasing number of mutations that are necessary for diagnosis, it is becoming impractical to do a single gene testing analysis. Furthermore, multigene assessment is providing guidance for determining prognosis in many diseases. Since many of the diseases encountered in clinical practice show more than one molecular abnormality, we could adapt NGS based assays to have simultaneous detection of multiple somatic mutations in tens or hundreds of target genes that are associated with specific diseases. NGS assays are becoming cheaper, faster, and rapidly adopted by many laboratories and since there is an increasing number of actionable targets that could benefit from these assays. However, NGS technology requires highly complex bioinformatics and laboratory technology to develop a diagnostic pipeline. Currently, there are two commonly used NGS. Sequencing differs in these technologies but they use similar bioinformatics principles for determining abnormal sequences that are not matching reference sequences.

There are also three different enrichment methods used for NGS sequencing: Hybrid capture, Amplicons, and Anchored Multiplex polymerase chain reaction (1). However, until now there is no single database available for clinical annotations to determine the significance of variants. Moreover, these assays are expensive and reimbursement remains low. 

The introduction of NGS into clinical laboratories has significantly changed the use of molecular techniques. Testing for multiple genes in a panel using NGS platforms is more practical and economical (2,3). Furthermore, the cost of adding a new gene to the panel is relatively inexpensive, in comparison to performing multiple single-gene tests. The presence of specific genetic mutations could aid clinicians in 4 ways: 1) diagnostic algorithms, 2) prognosis assessment, 3) use of targeted therapy, and 4) monitoring treatment response and residual disease. Despite increased utilization, NGS assays remain challenging particularly for the identifications of variants without a known clinical significance. To address these concerns, new terms in NGS reports have been adopted: “actionable”, “potentially actionable”, “Variant of Undetermined Significance”, “likely benign” or “benign”.

## Mutation profiles in acute myeloid leukemia

**Figure 1:**
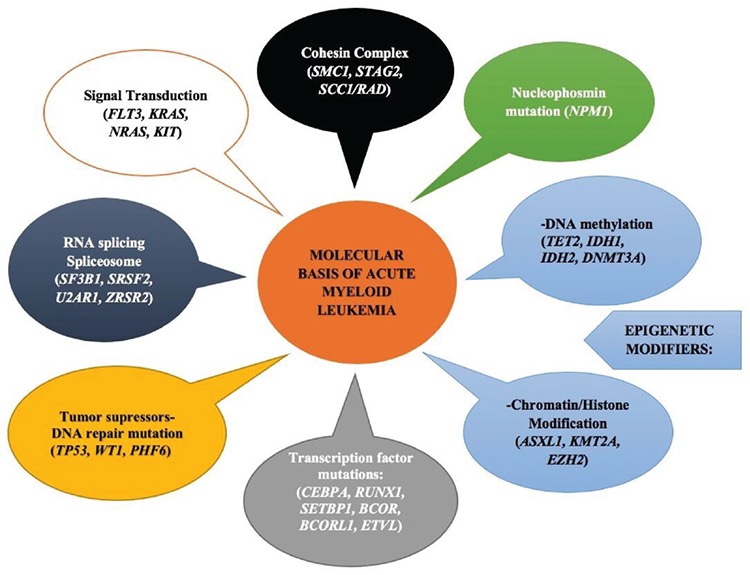
The most frequent gene mutations and cellular pathways in acute myeloid leukemia.

The Cancer Genome Atlas Network has many studies that report recurrent mutations and show a complex network of genetic mutations (4,5). Despite these exhaustive studies, there is no gene-specific diagnostic entity. However, there are some mutations leading to an aberrant activation of proteins that have a crucial effect on hematopoietic progenitor cell proliferation and differentiation. The mutations in AML involve epigenetic modifiers *(TET2, IDH1/IDH2, DNMT3A, ASXL1, KMT2A, EZH2),* activated signaling pathway *(FLT3, KRAS, NRAS, KIT),* tumor suppressor genes *(TP53, WT1),* RNA splicing *(SF3B1),* nucleophosmin mutation (NPM1), and genes coding for transcription-differentiation *(CEBPA, RUNX1)* (Figure 1) (6,7,8). Recurrently mutated genes in AML and MDS are listed in Table 1.

Recent studies have demonstrated that genes mutated in AML with a normal karyotype AML (NK-AML) share significant overlap with genes mutated in MDS (9,10). The World Health Organization (WHO) 2016 classification recognizes AML with mutated *NPM1* and AML with bi-allelic mutation of *CEBPA* as specific to AML classification categories and AML with mutated *RUNX1* as a provisional entity (8,9,10). The most frequently mutated genes in NK-AML occur in the following genes: nucleophosmin *(NPM1),* Fms-related tyrosine kinase 3 *(FLT3)* and DNA methyltransferase 3A *(DNMT3A),* Ten-eleven translocation-2 *(TET2),* isocitrate dehydrogenase 1 and 2 *(IDH1* and *IDH2),* CCAAT/enhancer binding protein alpha *(CEBPA),* NRAS, Additional sex comb-like 1 *(ASXL1),* WT1 and runt-related transcription factor-1 *(RUNX1)*. Mutations of *FLT3, DNMT3A,* and *NPM1* are often present concurrently, while other mutations of *NPM1, RUNX1, CEBPA,* and *TP53* are almost always mutually exclusive both at diagnosis and during disease transformation (11,12,13,14,15,16,17,18,19).

The WHO classification of AML includes many specific chromosomal abnormalities determined by karyotyping (11). The gene mutations recognized as specific entities by the 2016 WHO classification include *NPM1, CEBPA,* and *RUNX1* mutations. However, since the publication of this classification, it became very important to clinically recognize other pathogenic variants such as those involving *FLT3* *(ITD* and *TKD)* and *IDH1/2*. This can drastically change the clinical management of AML patients since the US Food and Drug Administration (FDA) has approved specific inhibitors for *FLT3* and *IDH2* positive AML (20,21).

The gene *NPM1* is the most frequently mutated gene in the adult type AML. It occurs approximately in 50 to 60% of cytogenetically normal AMLs and in 2 to 5% of MDS cases. It is often characterized by CD34 antigen negativity with a bright expression of CD33 and monocytic differentiation. *NPM1* mutations include small insertions (4-11bp in size), resulting in an aberrant cytoplasmic localization of the mutant protein nucleophosmin which can be detected immunohistochemically (21,22). *NPM1* mutations increase disease-free and overall survival in patients with AML. Secondary mutations [*FLT3-ITD, DNMT3A* (21), *IDH, TET2* mutations] are frequently accompanying these patients. *FLT3-ITD* mutations are observed in 40% of *NPM1* mutated AML patients. In general, *FLT3 ITD* positive patients without other molecular abnormalities show poor clinical outcome while *NPM1* positive patients have a better clinical response. Coexistence of *FLT3-ITD* mutation in *NPM1* positive patients is associated with a poor prognosis in young AML patients compared with patients with *NPM1 ^mut^ /FLT3 ^wild^* type (4,11,17,23).

Among transcription factor mutations in myeloid neoplasia, *CEBPA* and *RUX1* mutations are deemed important by the WHO due to their prognostic implications. CCAAT Enhancer Binding Protein-alpha *(CEBPA)* is a transcription factor responsible for promoting granulocytic maturation in regulating cellular function. *CEBPA* mutations are observed in approximately 10% of *de novo* AML patients and are commonly bi-allelic. The bi-allelic mutation is a favorable prognostic marker. Germline mutations of CEBPA predispose to the development of AML in young patients. The concurrence of *FLT3-ITD* mutation is an adverse prognostic finding in *CEBPA* mutated-AML patients (23). *RUNX1* encodes the DNA binding alpha subunit of the core binding factor *(CBFalpha)* that is involved in the normal differentiation of hematopoietic cells. *RUNX1* has also tumor suppressor properties and loss of function of the gene that contributes to tumorigenesis in myeloid cancers. *De novo* AML with mutated *RUNX1* in the absence of MDS related cytogenetic abnormalities appears to be a distinct entity with adverse outcome. *RUNX1* mutations are detected in approximately 4% to 16% of AML and 9% of MDS (11). They are commonly seen in older patients with NK-AML and MDS-associated AML. In several multivariate analyses, they have been associated with worse overall survival (23,24,25).

*FLT3* is a class III receptor tyrosine kinase coding for signal transduction gene. This receptor is important for proliferation and differentiation of hematopoietic stem cells. Its expression is downregulated during differentiation. *FLT3* mutations occur in 30% to 40% of AML patients with normal cytogenetics and 2% to 6% of MDS cases. Two types of mutations are observed in *FLT3* gene: *FLT3-ITD (internal tandem duplications)* mutations (with a frequency of 75% to 80%) and *FLT3-TKD (tyrosine kinase domain)* (with a frequency of 20% to 25%). FLT3 mutated patients show higher white blood cell count, loss of HLA-DR and CD34 antigen negativity, and impaired survival (26). AML patients with *FLT3-ITD* mutation appear to benefit from allogeneic stem cell transplantation. However, the prognostic effect of *FLT3-TKD* remains controversial (27). In 2017, the FDA approved treatment with midostaurin for newly diagnosed patients with FLT3-mutated AML due to the observation of a significant beneficial outcome (28,29). In MDS, *FLT3* mutations are observed in high-risk subgroups and are associated with complex karyotype (30,31). *NPM1* and *FLT3* mutations are found primarily in AML; however, the presence of these mutations in an MDS patient should alert the clinician about the risk of rapid progression to AML (22). Alterations on *FLT3* signaling pathway seems to be the most important prognostic factor for overall survival in AML patients younger than 60 years.

The other signal transduction genes are *KIT* mutations that are frequent in gastrointestinal stromal tumors, germ cell tumors, melanomas, and systemic mast cell diseases. They can be detected in approximately 6% of patients with newly diagnosed AML and 20% of AML patients with t(8;21) *RUNX1-RUNXT1* and 30% of AML patients with inv(16) or t(16;16) *CBFB-MYH11* (the core binding factor AMLs-CBF-AML). *KIT* mutation in AML increases the risk of relapse and worsens the good prognosis of CBF-AML (32,33,34,35,36).

There are a number of other genes playing a role in the pathogenesis of AML and appear to be important for overall survival despite not being recognized as specific entities. DNA methylation mutations and chromatin/histone modifications belong to epigenetic modifiers. *DNMT3A* encodes an epigenetic regulator and mediates *de novo* methylation of CpG dinucleotides and are seen in approximately 20% of AML patients with normal karyotype. This mutation tends to shorten overall survival (14,37). *TET2* is also involved in the epigenetic regulation of DNA methylation. *TET2* mutations occur in approximately 10% to 15% of AMLs. As *TET2* is a key regulator in hematopoietic stem cell renewal and differentiation, their mutations result in increased stem cell renewal and myeloid hyperplasia with impaired differentiation. These tend to have a poorer prognosis in NK-AML. *TET2* mutations predict the response to hypomethylating agents (4,38).

*IDH1/IDH2* mutations prevent the conversion of isocitrate to alpha-ketoglutarate in the Krebs cycle; create an oncometabolite that inhibits the function of *TET2. IDH1/IDH2* mutations often mutate with *TET2* and *WT1* mutations (39). Between 15% to 25% of AML and approximately 2% to 12% of MDS cases display *IDH* mutations (40). The prognostic significance of *IDH* mutations in AML is currently unclear. IDH2 inhibitor had been successful in the management of *IDH2* positive AML patients while IDH1 inhibitor is showing promising results in ongoing clinical trials (4,20,34,40).

Chromatin/Histone Modification mutations such as Additional Sex Coms Like Transcriptional Regulator 1 *(ASXL1)* mutations and Enhancer of Zeste 2 Polycomb Repressive Complex 2 Subunit *(EZH2)* mutations are a part of the epigenetic modifiers. *ASXL1* gene is involved in the epigenetic regulation of gene expression. They are uncommon in *de novo* AML (6.5%), but more frequent in AML arising from a prior myeloid neoplasm (up to 30%) (41,42,43). These mutations are associated with shorter overall survival and resistance to chemotherapy. *KMT2A: lysine(K)-specific methyltransferase 2A (KMT2A/MLL)* gene plays a role in hematopoiesis and cell differentiation. *KMT2A* mutations co-occur with an additional gene mutation *(IDH2/DNMT3A/U2AF1/TET2)*. This mutation is frequently associated with trisomy 11 (90%) and a poorer prognosis (17,44,45).

TP53 protein is a transcription factor and tumor suppressor gene that determines whether the cell undergoes repair, senescence or apoptosis. Although somatic *TP53* mutations are frequently seen in 50% of solid tumors, they are uncommon in *de novo* AML (5% to 18%) and they are associated with secondary or therapy-related AMLs. *TP53* mutations often coexist with complex karyotypes, chemotherapy resistance, and reduced overall survival compared with *TP53 ^wild^* AML patients. *TP53 ^mut^* AML patients have also high relapse rates after stem cell transplantation (46,47,48). Despite these findings, a recent study indicated that AML patients with cytogenetic abnormalities associated with unfavorable risk, *TP53* mutations, or both had a favorable clinical response to decitabine (49,50).

**Figure 2:**
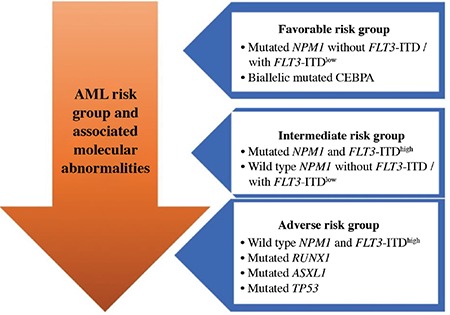
AML risk categories and associated molecular abnormalities. (FLT3-ITD ^low^ : FLT3-ITD/wild type allelic ratio <0.5; FLT3-ITD ^high^ : FLT3-ITD/wild type allelic ratio >0.5) AML: acute myeloid leukemia

Figure 2 represents these risk categories associated with distinct molecular mutations that should be screened by NGS in AML patients.

For the monitoring of minimal residual disease (MRD) in AML; NGS based technologies can be used besides multiparameter flow cytometry (MFC). MFC is a more reliable method for monitoring remission than conventional morphology-based assessment in AML patients; however, MFC lacks sensitivity for detecting residual disease. Therefore, a combined result of sequencing and flow cytometry detects residual disease more specifically. A recent study about molecular MRD in AML showed that the detection of molecular MRD is associated with a significantly higher relapse rate (51). During the complete remission, persistent mutations associated with clonal hematopoiesis *(DNMT3A, TET2,* and *ASXL1* referred as *DTA* mutations) and non-*DTA* mutations can be detected by NGS. While *DTA* mutations do not have prognostic value for MRD, non-*DTA* mutations (such as mutations in *TP53, IDH1, IDH2* and genes related to the Ras pathway) with a higher allele frequency can predict relapse rates (51,52).

Among AML with myelodysplasia-related changes (AML-MRC) and therapy-related AML (t-AML), mutations in splicing factors genes such as *SRSF2, SF3B1, U2AF1, ZRSR2,* mutations on chromatin/histone modifications like *ASXL1, EZH2, BCOR, STAG2,* and *TP53* mutations are common (53). *NPM1* mutations in the absence of *FLT3-ITD* and double *CEBPA* mutations are fairly uncommon in AML-MRC and are associated with favorable overall survival. Patients in this category with *TP53* mutations had a poor prognosis regardless of age (46,54,55,56). After treating patients with alkylating agents/ionizing radiation, patients often have increased blasts with associated multilineage dysplasia that cause similar mutation profiles like in AML-MRC. Mutations of *TP53* can be seen in as many as 50% of t-AML cases with worse survival (11,53,57,58). Following a topoisomerase II inhibitor therapy, 20 to 30% of patients develop overt acute leukemia without a preceding myelodysplastic phase. These patients often show balanced chromosomal translocations involving 11q23 *(KMT2A/MLL)* or 21q22 *(RUNX1)* and are associated with monoblastic or myelomonocytic morphology (11,53,59,60). Mutations in *TP53, TET2,* and *PTPN11* (protein tyrosine phosphatase, non-receptor type 11), *IDH1/2, NRAS* are frequent in t-AML, but *FLT3* and *NPM1* mutations are less frequent than de novo AML (58,61).

## Mutation profiles in Myelodysplastic Syndrome

Approximately half of the myelodysplastic syndrome is associated with recurrent cytogenetic abnormalities such as -5/del(5q), -7/del(7q), +8del(20q) or complex karyotypes (62). Targeted panels and whole-genome NGS assay detect somatic mutations in up to 90% of MDS patients (63,64,65). Some of the mutations overlap with those observed in AML, none of these mutations are specific for MDS diagnosis. However, having multiple genetic abnormalities with high variant allele frequency is starting to be recognized for its supportive evidence of evolving MDS (66).

The most frequently mutated genes in MDS are SF3B1 (~25%), *TET2* (~25%-20%), *ASXL1* (~15%), *SRSF2* (~15%), *RUNX1* (~15%-10%), *DNMT3A* (~15%-10%) (23). Mutations in *ASXL1, TP53, EZH2, ETV6,* and *RUNX1* are also found to be recurrent in MDS patients and predict a poor overall survival (41). *DNMT3A* mutations occur early in the course of MDS and suggest an early genetic event in leukemogenesis. Patients with *DNMT3A* mutations have a worse overall survival and more rapid progression to AML. They are associated with mutations in *FLT3* *(ITD* or *TKD),* *NPM1, IDH1* genes (67). *TP53* mutations in MDS are associated with the very aggressive disease. Even in low-risk MDS with del(5q), the presence of a *TP53* mutation at low frequency is correlated with a higher rate of leukemic transformation and poor response to lenalidomide (11,68).

Mutations in splicing factors are commonly detected in MDS patients with a frequency of 50%. The most common of these mutations are *SF3B1, SRSF2, U2AF1,* and *ZSR2* mutations. *SF3B1* mutations are the most common among splicing mutations in MDS. These mutations are highly correlated with the presence of ring sideroblast (RS). They are observed in 20% to 28% of MDS, more frequent in MDS with RSS and the frequency of *SF3B1* mutation in AML is <5% (11,23,69,70,71). Mutations in Cohesin Complex genes *(SMC1, SMC3, SCC1/RAD, STAG2)* can be germline resulting in Congenital Malformation syndrome. These mutations can be detected in 10% to 25% of MDS and 10% to 15% of *de novo* AML (17,69).

Nearly all cases of MDS show at least one mutation and demonstrate that specific mutation with a targeted NGS panel is highly useful for the diagnosis of MDS. However, determination of mutational burden/variant allele frequency (VAF) (usually >25% for MDS) is critical for the diagnostic assessment to distinguish MDS from clonal hematopoiesis of indeterminate potential (CHIP) or clonal cytopenia of undetermined significance (CCUS). CHIP is the proposed term for healthy individuals, lacking hematological malignancy or clonal disorder, but carrying a hematological somatic mutation. Key findings in CHIP are single gene mutations (mostly *DNMT3A, TET2* or *ASXL1* mutation), increased risk of developing hematological malignancy, and increased rate of cardiovascular mortality regardless of their cancer risk. The single mutation burden in CHIP has a VAF more than 2% but typically not exceeding 20%-30%, which allows the distinction from underlying dysplastic process (3). Patients who are not meeting the WHO defined criteria for a hematologic neoplasm, but show a clonal mutation with unexplained cytopenia, are referred to as CCUS. The mutation spectrum of CCUS patients is similar to mutations seen in MDS. Mutations in spliceosome genes *(SF3B1, SRSF2, U2AF1, ZRSR2)* have the highest predictive value for CCUS (72). Co-mutations of *DNMT3A, TET2,* and *ASXL1* genes remain also common in CCUS. There are no clear recommendations to follow up CCUS patients. However, cytopenia, mutation burden, number and type of the mutations or the detection of additional mutations in the follow-up of a patient with suspected MDS appear to have a course similar to MDS (72,73,74,75,76).

## Mutation profiles in myeloproliferative and myelodysplastic/myeloproliferative neoplasms

Disorders altering the myeloid elements include the following disorders: Polycythemia vera (PV), essential thrombocythemia (ET), primary myelofibrosis (PMF), chronic myelogenous leukemia (CML), chronic neutrophilic leukemia (CNL), hypereosinophilic leukemia/CEL, CMML, and mast cell disease. Among CMPD (PV, ET, PMF, and CML), molecular markers have been essential for diagnosis. Recurrent somatic mutations in *BCR-ABL2* negative MPNs and MDS/MPNs are listed in Table 2.

The most frequent mutation in *BCR-ABL2* negative MPN, *JAK2* exon 14 (V617F) mutations are observed in 95% of PV patients (77). The majority of remaining PV patients may harbor a mutation in *JAK2* exon 12 (missense mutation, deletion, insertion). *JAK2* (V617F) mutation occurs in approximately 50-60% of ET or PMF patients. Homozygous mutations of *JAK2* (V617F) (with a VAF value >50%) are more common in PV than in ET (78,79). *JAK2* (V617F) mutation may not be present in half of the MPN patients. This type of mutation has rarely been reported in patients with MDS/MPN; however, they are found in patients with MDS, CMML, atypical CML, and *de novo* AML (approx. <5%) and MDS/MPN-RS-T (approx. 50%). In PV and ET; *JAK2* (V617F) allele burden is also associated with a more aggressive behavior. Thrombotic events and fibrotic transformation are more common in patients with a high *JAK2* (V617F) allele burden (higher *JAK2* VAF). These findings suggest that monitoring *JAK2* (V617F) allele burden could be useful to identify patients at higher risk of myelofibrotic transformation. *JAK2* mutation in PMF is associated with intermediate prognosis when compared with *CALR* mutation (3,11,17,23,80).

Patients with ET have mutations in the *CALR* gene that encodes the endoplasmic reticulum and are associated with chaperone calreticulin at a frequency of 20% to 25%. CALR mutations are frequently seen in ET and PMF patients with *JAK2 ^wild^* and *MPL ^wild^* type. They are rarely detected in PV, CMML, MDS/MPN patients. *CALR* mutations are not seen in AML, mastocytosis, lymphoid neoplasia and solid tumors (81,82). *CALR* mutated MPNs demonstrate distinct features; such as younger age group, lower hemoglobin levels, higher platelet counts, decreased risk of thrombosis, and improved overall survival compared to patients with *JAK2* or *MPL* mutations (83,84). In ET and PMF patients, *CALR* show 2 different mutations: type-I that typically has 52-bp deletion (p.L367fs*46), and  type-II characterized by 5-bp TTGTC insertion (p.K385fs*47). Type I CALR mutation is detected more frequently in PMF than ET. Type II *CALR* mutations tend to show leukemic transformation (namely increased leucocyte count and higher circulating blasts) than type I *CALR* mutation (81,85,86). Platelet count is significantly higher in type II vs type I *CALR ^mut^* ET patients, but no difference was noted in thrombosis-free survival in a study with a large cohort of ET patients. However, there is a significant survival difference in the setting of PMF, as CARL type-I positive PMF patients do much better than *CALR* type-II or *JAK2* (V617F) positive PMF patients (84).

**Figure 3:**
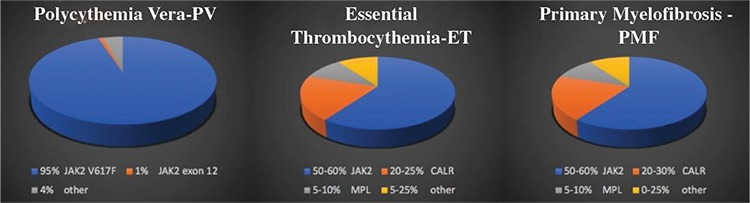
Distribution of mutation profiles in myeloproliferative neoplasm. ET: essential thrombocythemia; PMF: primary myelofibrosis; PV: policytemia vera

The activating mutation on codon W515 of *MPL* gene is seen in 5-10% of ET and PMF patients who are negative for *JAK2 *(V617F) (87). Patients that are *JAK2* ^wild^  and *CALR* ^wild^ , but harboring ET or PMF clinical features should be screened for *MPL* mutation. *MPL* mutated patients have increased the risk for thrombotic episodes and transfusion requirement compared with *JAK2* ^mut^  and *CALR* ^mut^  patients (80,88,89). Patients with PMF have mutations for *JAK2* in 60%, *CALR* in 20-30% and *MPL *in5-10% of the cases (Figure 3). The remaining 10% to 15% of patients with ET or PMF patients have none of the above-mutated genes and these are named “triple negative” (85,90,91). This is a heterogeneous category and some of the ET patients may have rare non-canonical mutations in *JAK2,* *MPL, *and* SH2B3*; however, the large majority remain without a specific mutation. For “triple negative” PMF patients, a detailed search for the mutation profiles regarded as a marker of clonality (such as *ASXL1,* *EZH2,* *TET2,* *IDH1/IDH2,* *SRSF2,* *SF3B1)* is currently recommended (77). “Triple negative” patients tend to show inferior outcome compared to *JAK2* ^mut^ */CALR* ^must^  and *MPL* ^mut^  CMPD patients. The presence of at least one of the 3 variants/mutations *(ASXL1, SRSF2,* and *IDH2) *is associated with inferior overall and myelofibrosis-free survival in patients with PV. *SH2B3, IDH2, U2AF1, SF3B1, EZH2, *and* TP53 *mutations are identified as significant risk factors for inferior overall, myelofibrosis-free survival, and leukemia-free survival in patients with ET (85).

Recent studies about leukemic transformation in MPNs show *ASXL1,* *SRSF2,* *IDH1,* *IDH2,* *RUNX1* mutations have been associated with leukemic transformation in PMF, whereas *SRSF2,* *IDH2* mutations in PV and *TP53,* *EZH2* mutations in ET predicts more leukemic transformation (92). These findings show that the mutation spectrum in primary NK-AML is different from secondary AML. Recurrent somatic gene mutations are observed in up to 90% of CMML patients. *TET2 *(~60%), *SRSF2 *(~50%), *ASXL1 *(~50%), *RUX1,* *NRAS,* and *TP53* are the most frequent mutations. However, these mutations can also be encountered in healthy aging people such as CHIP with a single mutation of a VAF <20-30%. Therefore, a low VAF of these mutations should not be used as a definitive diagnosis for CMML (23). *ASXL1* mutations are associated with a higher white blood cell count, lower hemoglobin, extramedullary disease, and an abnormal karyotype. *ASXL1* mutations lead to the aggressive clinical course in CMML patients (93,94). The *JAK2* mutation also occurs in CMML patients sharing some mutual features with *JAK2* ^mut^ MPNs. These cases predict more reticulin fibrosis, occasional megakaryocytic clustering with atypia, and erythroid and megakaryocytic hyperplasia (95). In case of presence, *NPM1* ^mut^ AML with monocytic differentiation should be suspected. Variable results are also expected on *JAK* ^mut^ CMML patients treated with JAK2 inhibitors (11,96,97).

Juvenile myelomonocytic leukemia (JMML) patients harbor also clonal cytogenetic abnormalities. The commonly mutated genes are *KRAS/NRAS,* *NF1,* *PTPN11,* *CBL,* which encode proteins of the Ras oncogene pathway. *PTPN11* mutations are the most frequent alterations (~35%). Somatic *NRAS *and *KRAS* mutations occur in 20-25% of JMML cases. Germline mutations in *CBL* and *NF1* genes are also frequent and are associated with Noonan Syndrome-like disorder and neurofibromatosis type-I on JMML patients, respectively (98,99).

*SETBP1* and ethanolamine kinase 1 *(ETNK1)* mutations are relatively common in atypical CML (aCML). *CSF3R* mutation is frequently associated with CNL, this type of mutation is uncommon (<10%) in aCML and rarely encountered in CMML and AML cases (100,101). Correlation of *CSF3R* mutation with CNL has been included in the diagnostic criteria in the revised 2016 WHO classification (10,21). In myelodysplastic/myeloproliferative neoplasm with ring sideroblasts and thrombocytosis (MDS/MPN-RS-T), *SF3B1* mutations are pesent in 70-90% of cases. They are frequently commutated with *JAK2 *(V617F) (50-65%) and less frequently with *CALR*/*MPL *(11,23,70,71).

Among myeloid neoplasms with eosinophilia; CEL-NOS, idiopathic hypereosinophilic syndrome, myeloid/lymphoid neoplasms with eosinophilia, and gene rearrangement are considered in the revised 2016 WHO classification. However, hypereosinophilia can be encountered in systemic mastocytosis, CML, other types of MPNs, MDS/MPNs, AML, MDS and T cell lymphoproliferative disorders. Once, cytogenetically *PDGFRA,* *PDGFRB,* *FGFR1* rearrangements, and *PCM1-JAK2* fusion and other causes of hypereosinophilia are excluded, NGS should be performed for the patients with eosinophilia.

Lastly, mast cell diseases show point mutations of the *KIT* gene (D816V) in 95% of cases. Although the presence of D816V mutations is important at diagnosis for systemic mastocytosis, a minority of patients harbor mutations on exon 17 or other exons (102) Additionally; *TET2,* *ASXL1,* *RUX1,* *SRSF2,* and *JAK2* mutations can be seen on the patients with mastocytosis (103,104).

We outlined some of the common genes that are important to assess in various hematologic neoplasms with emphasis on myeloid neoplasia. Despite the significant progress in lymphoid neoplasms, particularly for understanding its pathogenesis, NGS is no ready to be integrated into routine clinical testing. Most of the NGS panels offered for assessment of myeloid neoplasms contain between 30-60 targeted genes. The market for NGS-based diagnostic procedures is rapidly growing, therefore it is impossible to list all the available tests. However, based on current literature, a panel with the following genes will most likely be informative in clinical assessment for most of the myeloid neoplasms: *FLT3, NPM1, CEBPA, TP53, IDH1/2, DNMT3A, TET2, CSF3R, SRSF2, KIT, NRAS, RUNX1, WT1, ASXL1, SF3B1* for AML; *NRAS, KRAS, IDH1/2, TET2, EZH2, ASXL1, RUNX1, TP53, DNMT3A, SF3B1, U2AF1, ETV6 for MDS; JAK2, CALR, MPL, IDH1/2, ASXL1, TET2, EZH2, SRSF2, SF3B1, TP53*for MPN; *TET2, SRSF2, ASXL1, RUNX1, NRAS, TP53* for CMML; *SF3B1* for MD/MPN RS-T; *CSF3R* for CNL, *SETBP1* for aCML, *KIT (D816V)* for mast cell disease.

In conclusion; the progress of NGS and widen utilization on hematologic malignancies will facilitate a more precise diagnosis and treatment for patients with hematologic neoplasms. AML, MDS, and MPNs are characterized by morphologic or phenotypic similarities but these disorders harbor different mutations types with different prognostic implications. Myeloid neoplasms that lack a cytogenetic alteration but show a somatic mutation can be diagnosed by the above-mentioned mutation profiles. These mutations can also facilitate new novel therapeutic agents. In the coming years, it appears that major efforts with more comprehensive high throughput NGS sequencing and inclusion of genomic data in clinical management will enable the delivery of precision medicine.

## Figures and Tables

**Table 1:**
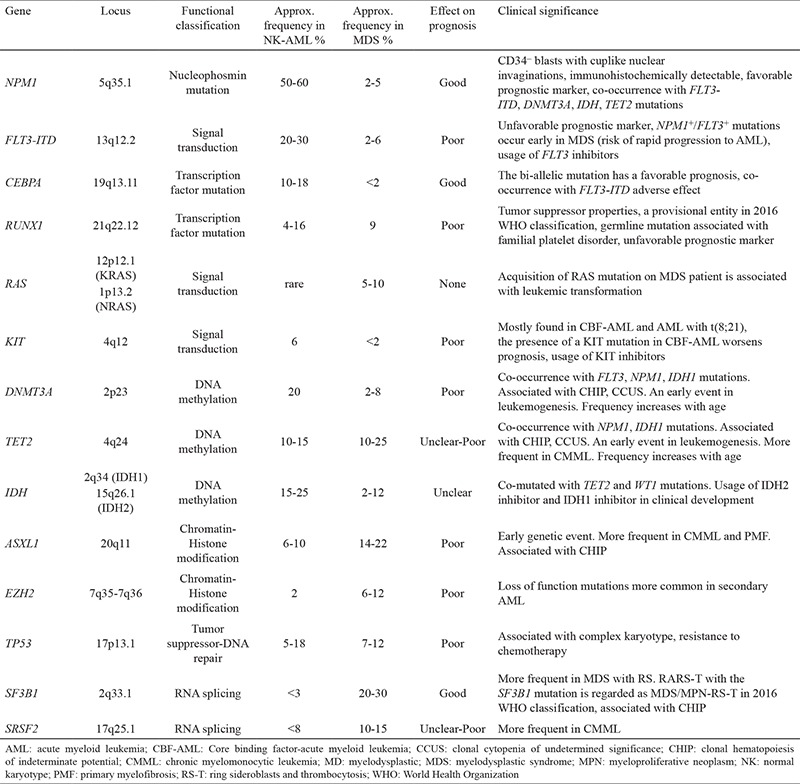
Common gene mutations in AML, MDS, and their clinical significance

**Table 2:**
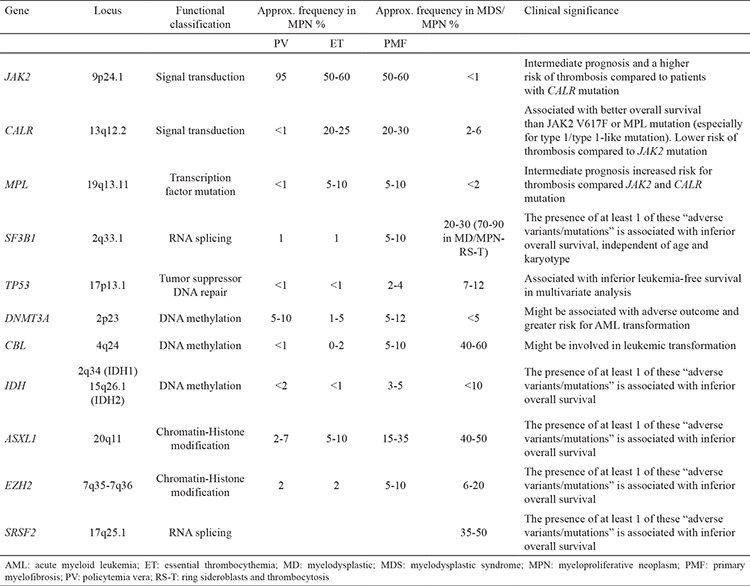
Common gene mutations in MPN, MDS/MPN, and their clinical significance
